# Another Cause of Acute Cardiogenic Shock

**DOI:** 10.36660/abc.9453

**Published:** 2019-12

**Authors:** Gonçalo Morgado,, Filipe Gonzalez,, Ana Alves Oliveira,, Antero Fernandes

**Affiliations:** 1 Hospital Garcia de Orta, Almada - Portugal

**Keywords:** Takotsubo Cardiomyopathy/complications, Heart Failure, Pheochromocytoma, Respiratory Insufficiency, Shock, Cardiogenic

## Clinical Case

A 64-year-old woman with a history of Diabetes and Hypertension was admitted to the emergency department with acute dyspnea and chest pain. Physical examination revealed hypertension, sinus tachycardia (150 bpm), acute pulmonary edema and poor extremity perfusion. In the context of respiratory failure and hemodynamic instability, she required invasive mechanical ventilation. Blood works showed increased levels of hs-TroponinT (rising from 800 to 1600 ng/L) and the ECG showed poor R wave progression and incomplete right bundle branch block. The echocardiogram revealed a hypertrophied left ventricle, with severe systolic dysfunction and akinetic apical and middle segments. Considering the possibility of acute coronary syndrome, the patient was referred for emergent coronary angiography, which revealed normal coronary arteries. She was admitted to the intensive care unit with the presumed diagnosis of Takotsubo cardiomyopathy. Through the rest of the day, she presented with fluctuating blood pressure and required elevated levels of positive end-expiratory pressure due to pulmonary edema. Despite an apparent favorable evolution, she suddenly developed asystole, refractory to resuscitation efforts, dying less than 24 hours after admission.

### Post-mortem examination

Macroscopic examination revealed: myocardium with softened hyperemic anterolateral wall, suggestive of myocardial infarction; mild pericardial effusion; bilateral pulmonary congestion with hepatization of the lung basal lobes; left retroperitoneal mass, with 10x7x5 cm, with a cystic appearance and a necrotic core, located above the left kidney.

**Figure 1:**
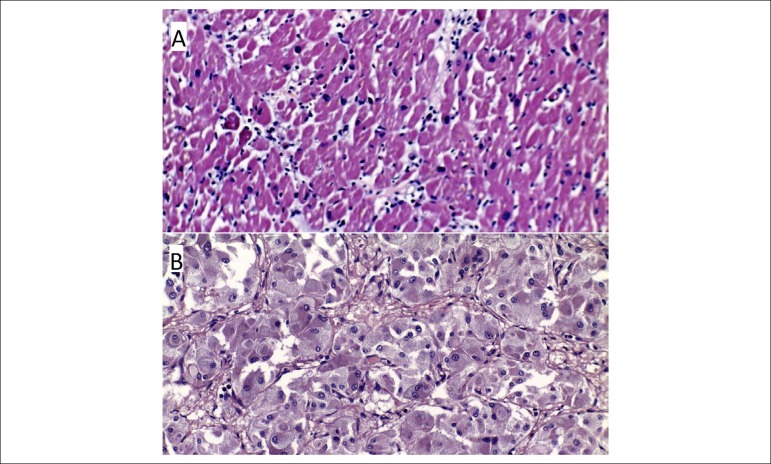
A) Histopathology specimen of the heart, haematoxylin and eosin stain: necrotic myocardium with inflammatory cells infiltrate. B) Histopathology specimen of a friable mass adjacent to the left adrenal gland, haematoxylin and eosin stain: nests of chromaffin tumour cells, with numerous membrane-bound granules, surrounded by a fibrovascular stroma.

Microscopic examination revealed: necrotic myocardium with inflammatory infiltrate Figure 1A), with no evidence of coronary artery disease; lungs with extensive alveolar edema and passive vascular congestion; left suprarenal gland tumour consistent with pheochromocytoma Figure 1B), with intratumoral hemorrhage and necrosis.

Anatomopathological diagnosis: left suprarenal pheochromocytoma; catecholamine-induced cardiomyopathy; acute pulmonary edema.

## Comments

In this case, the patient developed progressive ventricular dysfunction in the context of stress cardiomyopathy, leading to low cardiac output and cardiogenic shock. The pathology examination revealed a pheochromocytoma. This rare neoplasia has been described in the literature as a possible cause of stress cardiomyopathy due to an excess of circulating catecholamines, causing acute heart failure^1^ or cardiogenic shock.^2^

Contemporary literature reviews have found a higher complication rate for pheochromocytoma-induced stress cardiomyopathy when compared with idiopathic Takotsubo cardiomyopathy. Patients with pheochromocytoma are more likely to develoP cardiogenic shock (34.2% vs. 4.2%)^3^ and less likely to recover left ventricular function on follow-uP (40.8% vs. 64.9%).^4^ In such patients, circulatory support with extracorporeal membrane oxygenation has been demonstrated as feasible but is still associated with a significant mortality rate.^5^

The case is a reminder that pheochromocytoma should be a differential diagnosis in the context of stress cardiomyopathy, particularly in patients presenting with cardiogenic shock.
